# A Systematic Review of Health System Barriers and Enablers for Antiretroviral Therapy (ART) for HIV-Infected Pregnant and Postpartum Women

**DOI:** 10.1371/journal.pone.0108150

**Published:** 2014-10-10

**Authors:** Christopher J. Colvin, Sarah Konopka, John C. Chalker, Edna Jonas, Jennifer Albertini, Anouk Amzel, Karen Fogg

**Affiliations:** 1 Centre for Infectious Disease Epidemiology and Research (CIDER), Division of Social and Behavioural Sciences, School of Public Health and Family Medicine, University of Cape Town, Medical School Campus, Cape Town, South Africa; 2 Center for Health Services, Management Sciences for Health, Arlington, Virginia, United States of America; 3 Center for Pharmaceutical Management, Management Sciences for Health, Arlington, Virginia, United States of America; 4 United States Agency for International Development (USAID)/Africa Bureau, Washington, District of Columbia, United States of America; 5 USAID/Bureau for Global Health (BGH)/Office of HIV/AIDS, Washington, District of Columbia, United States of America; 6 USAID/BGH/Office of Health, Infectious Diseases and Nutrition, Washington, District of Columbia, United States of America; Medical University of Vienna, Austria

## Abstract

**Background:**

Despite global progress in the fight to reduce maternal mortality, HIV-related maternal deaths remain persistently high, particularly in much of Africa. Lifelong antiretroviral therapy (ART) appears to be the most effective way to prevent these deaths, but the rates of three key outcomes—ART initiation, retention in care, and long-term ART adherence—remain low. This systematic review synthesized evidence on health systems factors affecting these outcomes in pregnant and postpartum women living with HIV.

**Methods:**

Searches were conducted for studies addressing the population of interest (HIV-infected pregnant and postpartum women), the intervention of interest (ART), and the outcomes of interest (initiation, adherence, and retention). Quantitative and qualitative studies published in English since January 2008 were included. A four-stage narrative synthesis design was used to analyze findings. Review findings from 42 included studies were categorized according to five themes: 1) models of care, 2) service delivery, 3) resource constraints and governance challenges, 4) patient-health system engagement, and 5) maternal ART interventions.

**Results:**

Low prioritization of maternal ART and persistent dropout along the maternal ART cascade were key findings. Service delivery barriers included poor communication and coordination among health system actors, poor clinical practices, and gaps in provider training. The few studies that assessed maternal ART interventions demonstrated the importance of multi-pronged, multi-leveled interventions.

**Conclusions:**

There has been a lack of emphasis on the experiences, needs and vulnerabilities particular to HIV-infected pregnant and postpartum women. Supporting these women to successfully traverse the maternal ART cascade requires carefully designed and targeted interventions throughout the steps. Careful design of integrated service delivery models is of critical importance in this effort. Key knowledge gaps and research priorities were also identified, including definitions and indicators of adherence rates, and the importance of cumulative measures of dropout along the maternal ART cascade.

## Background

Despite global progress in the fight to reduce maternal mortality over the last 20 years, HIV-related maternal deaths remain persistently high [1], [2], with approximately 24 percent of deaths in pregnant and postpartum women in sub-Saharan Africa attributable to HIV [3]. Lifelong antiretroviral therapy (ART) appears to be the most effective way to prevent HIV-related maternal mortality [4]. Although ART is increasingly available in the public sector health services of most countries, many pregnant or postpartum women continue to struggle to access treatment, and the rates of initiation, retention in care, and long-term adherence remain troublingly low [5], [6].

This is surprising not only because of the increasing availability of ART, but also because pregnant women represent a sector of the population that is among the most consistently engaged with the health system. Why are HIV-infected pregnant and postpartum women continuing to die from HIV-related causes when effective ART and prevention of mother-to-child transmission (PMTCT) programs are increasingly available? What prevents these women from accessing treatment during and after pregnancy?

There is a large and growing literature on health systems barriers to and enablers of ART. This literature has identified a wide range of key factors, including long waiting times, transport costs, fears of confidentiality breaches, poor staff attitudes and fragmented service delivery platforms [7]–[10]. Many of these factors also affect pregnant or postpartum women who need ART. In this review, we examined the challenges specific to providing ART to pregnant women 1) who may or may not already be engaged with other health services (i.e., antenatal care or PMTCT programs) and 2) who may be more vulnerable or have specific health needs associated with their pregnancy and up to one year after delivery.

This review is one of three systematic reviews that together consider evidence around efforts to reduce mortality among HIV-infected pregnant and postpartum women. The current review examines the health system (or supply-side) barriers to and enablers of three key outcomes among pregnant and postpartum women: ART initiation, retention and adherence and evidence on health system interventions that may facilitate access to maternal ART. A second review synthesizes evidence on demand-side barriers and enablers to ART [11]. ‘Demand-side’ factors include those individual, interpersonal, community, and structural factors outside of the health system that influence an HIV-infected woman's ability to initiate and adhere to ART. The third review assesses the evidence on the effectiveness of various interventions to reduce mortality among HIV-infected pregnant and postpartum women [12].

## Methods

### Review Design

We conducted a systematic review of qualitative and quantitative evidence regarding health system barriers and enablers of access to ART for HIV-infected pregnant and postpartum women. For the analysis of study findings, we used a four-stage narrative synthesis design [13] that was informed by the World Health Organization (WHO) health systems strengthening [14] and Supporting the Use of Research Evidence (SURE) frameworks [15], with comparative case analyses across a range of possible contextual factors.

### Study Eligibility

#### Inclusion Criteria

To maximize the breadth of data included in the review, we included any study that reported empirical qualitative or quantitative findings relevant to the review question. Studies from low- and middle-income countries (LMICs), as well as high-income countries, were included, as were studies conducted in community, primary care or tertiary care settings. Due to time and resource constraints, we only included studies written in English. To maximize the relevance of the study findings to current maternal ART policy and practice, we only included studies published between January 1, 2008 and March 26, 2013.

#### Exclusion Criteria

We excluded studies that focused only on PMTCT vertical transmission outcomes or that included only PTMCT or maternal ART program outcomes but did not identify or discuss the related health systems barriers or enablers. We also excluded studies on broader cohorts of people with HIV (e.g., all adults on ART) if barriers and enablers specific to pregnant or postpartum women could not be distinguished in the findings.

### Search Strategy and Selection Process

#### Search strategy

To identify eligible studies, we first searched for peer-reviewed publications through the PubMed and Social Science Citation Index databases. For the database searches, we used variations of search terms for 1) the *population of interest* (pregnant or postpartum women with HIV, 2) the *intervention of interest* (antiretroviral therapy) and 3) the *outcomes of interest* (initiation, adherence, retention). A full search strategy for one of the database searches can be found in the Supporting Information. Additional studies were identified through a gray literature search of conference abstracts, program reports, and government documentation related to ART for HIV-infected pregnant or postpartum women. A list of websites searched for gray literature can be found in the Supporting Information.

#### Study selection

Studies were selected for review in two stages. First, three review authors independently assessed the first 100 abstracts retrieved from PubMed. Each reviewer's list of selected articles and accompanying rationale was compared with the list from the other reviewers; discrepancies were discussed and resolved. Inclusion and exclusion criteria were refined and clarified during this process.

### Quality Assessment and Data Extraction

#### Characterizing the evidence base

Given the diversity of study designs included and the difficulty of comparing study quality assessments across widely varying study types, we addressed study quality in two separate steps. First, we developed an overview of key characteristics of the evidence base by summarizing several variables, including study design, sample size, geographic region, healthcare setting, and risk of bias (see Supporting Information). We ranked each included study as low, medium or high with respect to overall risk of bias, based on its sample size, selection criteria, sampling procedure and data analysis method. Rankings of study quality were based on criteria relevant to each study design and were justified with a short narrative (see Supporting Information). This provided an overview of the quality of the existing evidence base, as represented by the included studies. No studies were excluded on the basis of the quality assessment. Rather the quality assessment process was used to identify weaknesses in study methodologies and to strengthen interpretation and assessment of study findings.

In the second step, we used the summary of evidence-based characteristics to assess the strength and generalizability of the evidence underlying the key review findings. This step is described in more detail in the Data Synthesis sub-section below.

#### Data extraction and management

Following study selection, one review author (SK) extracted data from the studies using a standard template. Initial data extraction captured both the study characteristics (e.g., setting, participants, type of ART program reviewed) as well as key findings related to program outcomes and factors relating to initiation, retention, and adherence. A second author (CC) also reviewed the studies and extracted data relating to program outcomes and key health systems factors. Extracted findings from both authors were compared and discrepancies resolved.

### Data synthesis

The reviewers synthesized the evidence using a broadly comparative case-study approach informed by tools and techniques outlined in the narrative synthesis framework (see Supporting Information for a fuller explanation) [13]. The first step in this approach was developing a preliminary synthesis of the findings using an initial conceptual framework. To develop this framework, we drew from three sources: the WHO's health system ‘building blocks’ model [14], the SURE framework for identifying barriers and enablers to implementing health interventions [15], and a three-day scoping meeting held with all of the reviewers at the beginning of the project to refine the conceptual framework and ensure shared understanding of the key areas of interest for the review.

In the second step of the synthesis, two reviewers used this initial conceptual framework to guide the extraction of data and the identification of key barriers and enablers to maternal ART access. Emerging themes were discussed and refined throughout the data extraction process. One author synthesized these barriers and enablers into an overall framework of overarching themes and key findings that was then reviewed by the other reviewers for accuracy and comprehensiveness.

The third step explored whether certain independent factors related to the intervention or the context moderated the effect of the identified enablers and barriers. We examined differences between rural and urban settings, between routine and pilot/demonstration project settings, between various ART and PMTCT protocols and regimens, by co-morbidity, and by age group. We also explored the findings for other unanticipated associations.

The review findings were then organized into five themes, four descriptive and one related to interventions. Within each broad theme, we then identified one to four “key review findings.” In the narrative below, we also describe the individual findings that led to the development of that key finding.

In the fourth step of the narrative synthesis, we assessed the strength of the evidence for each of the “key review findings” and considered the extent to which these findings could be generalized. For each key review finding, we looked back at the studies that contributed to that finding and considered: 1) how strong the underlying study design was, 2) what the risk of bias was, 3) what level of detail and/or context was provided to enable interpretation, and 4) how frequently the review finding was found across the individual studies. We then ranked the strength of the evidence underlying each finding as strong, moderate or weak. We were also interested in the generalizability of the key review finding with respect to public sector health services in low and middle-income countries with high HIV prevalence. For each key review finding, we therefore asked two additional questions: 5) how many of the studies supporting this finding were conducted within existing services settings, and 6) how many came from LMICs with high HIV prevalence. Here too, we ranked generalizability as strong, moderate or weak.

In the summary table of key review findings (see below), we provide these rankings along with a very brief narrative justification for each. In the Supporting Information, we provide a fuller description of this method and justify our rankings by summarizing our answers to the above questions in more detail. While this assessment is narrative rather than quantitative, the fuller description of how the six questions were answered and the rankings were determined strengthens accountability and transparency and enables independent review of our assessment.

## Results

### Overview of Studies Included and ART Programs and Outcomes

**Figure 1:**
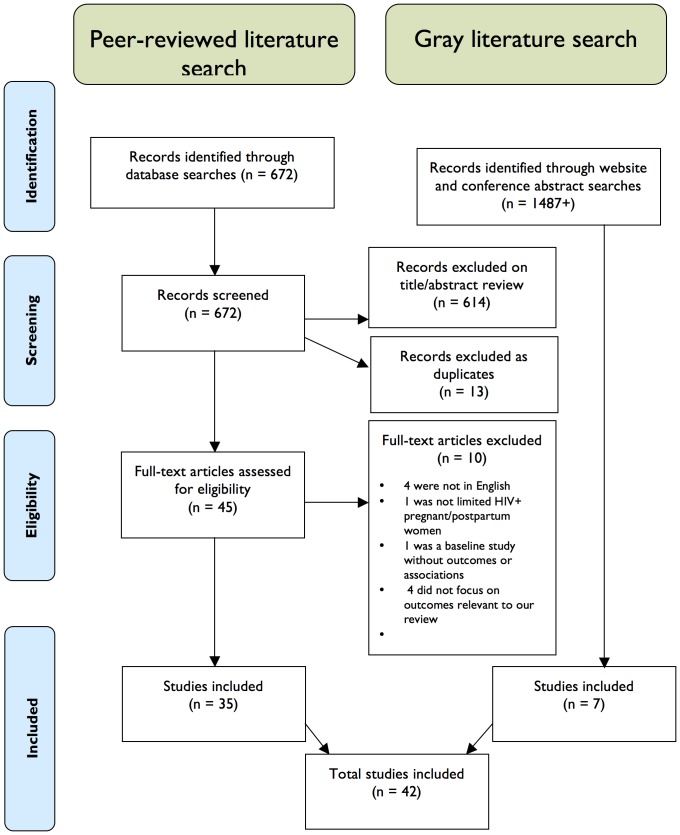
Flow Diagram for Study Search and Inclusion.

A total of 2,159 titles and abstracts were identified for screening; 42 papers and conference abstracts were included in the full review [16]–[57]. See Figure 1 for a flow diagram of the search and inclusion process and Table 1 for a summary of the studies included.

**Table 1:** Summary of Characteristics of Included Studies.

Characteristics		Number of studies
**Regions**		
	Sub-Saharan Africa	36
	Asia	2
	Latin America	3
	Europe/North America	1
	Middle East	0
**Geographic Setting**		
	Rural	15
	Urban	7
	Both	15
	Unclear	5
**Type of Facility**		
	Clinic	14
	District Hospital	6
	Tertiary Hospital	14
	All	2
	Unknown or N/A	6
**Study Designs** *		
	Retrospective Record Review	16
	Prospective Cohort	8
	Intervention/Evaluation	5
	Qualitative	13
	Unclear	1
**Sample Sizes**		
	*From small qualitative through to large record reviews*	Range: 7–663,603 Median: 396
**Primary Outcome of Interest** *		
	Initiation	30
	Retention	10
	Adherence	9
**Explicit Intervention Tested**		
	Yes	12
	No	30

*Sum exceeds the total number of studies because some studies were counted in multiple sub-categories.

The vast majority of the studies included were conducted in sub-Saharan Africa, in rural or both rural and urban settings, used clinical epidemiology study designs, and addressed ART initiation as the primary outcome of interest. Study participants included HIV-infected pregnant and/or postpartum women and/or health care providers delivering antenatal care (ANC), ART and/or PMTCT. A few studies included partners and/or family members of HIV-infected pregnant or postpartum women.

Most of the studies were from service delivery settings that used triple therapy antiretroviral (ARV) prophylaxis for the mother, or initiation of lifelong ART if CD4 <350. Eight studies were from settings using a single-dose nevirapine (sdNVP) protocol and two studies reported results from programs using the Option B+ protocol (initiation of lifelong ART regardless of CD4 level). Only 12 studies formally tested an intervention related to maternal ART. A table listing the characteristics of the included studies can be found in the Supporting Information.

Given the importance of maintaining adherence to treatment regimens, only nine (21 percent) of the included studies assessed either short- or long-term ART adherence, with three studies conducted in settings that used the outdated sdNVP protocol. To measure adherence, the studies used a variety of indicators as there was a lack of a uniform definition. Table 2 summarizes the wide variety of indicators used in the studies to measure adherence.

**Table 2:** Indicators of Adherence Used in the Studies.

Study	Adherence Indicator
Awiti [16]	Measure of adherence and type (short/long term, etc.) not noted; duration on ART was between 1 and 6 years, self-reported
Ayuo [17]	Measure: disengagement (early disengagement = no contact for any period of 30 or more consecutive days between first antenatal visit and delivery; late disengagement = no contact during the 30 days prior to delivery); self-reported adherence
Delvaux [22]	Adherence defined as mother-infant pairs who ingested SD-NVP at recommended time; non-adherence = not ingesting at all or at wrong time
Kasenga [26]	Unclear
Kirsten [27]	Adherence to combination prophylaxis for PMTCT (AZT at week 28, sdNVP) assessed drug collection, dispensing (pre and postpartum) and ingestion (during delivery/hospitalization)
Kuonza [28]	Adherence to sdNVP; non-adherence includes those who did not ingest or those that took at the wrong time; study also looks at infant adherence and combined maternal-infant adherence
Mellins [30]	Adherence based on self-report, having taken all doses in two prior days; also asked to report on last missed dose (past week, past two week, past month, past three months, more than three months ago, never)
Nassali [36]	Adherence to prenatal PMTCT program measured as proportion of mothers who honored appointments by end of 8 weeks postpartum
Peltzer [39]	Adherence to sdNVP; self-reported

### Overview of Health Systems Barriers and Enablers Identified

We divided the review findings into five descriptive themes: 1) models of care, 2) service delivery, 3) resource constraints and governance challenges, 4) patient/health system engagement, and 5) interventions to improve maternal ART outcomes. There are one to four “key findings” under each theme which are summarized in Table 3. The table also identifies the strength and generalizability of the evidence (See the Supporting Information for a fuller narrative justification of these assessments).

**Table 3:** Summary of Key Findings and Underlying Evidence.

MAJOR THEMES AND KEY FINDINGS	STRENGTH OF THE EVIDENCE	GENERALIZABILITY OF THE EVIDENCE
**Theme 1: Models of Care**		
a) Maternal ART services struggle to retain women in care and involve their partners, especially during the postpartum period and when women are ART-ineligible or have declined ART.	**Strong** Several high-quality studies with good detail reported this finding	**Strong** Reported in both a wide variety of clinical settings and geographic regions
b) Gaps between ANC/PMTCT and HIV services and dropout along the maternal ART cascade are persistent, widespread problems, even when models of care are designed to overcome typical access barriers such as vertical programs, physical distance, wait time, and fears of confidentiality.	**Strong** Detailed reporting in many studies, both quantitative and qualitative	**Strong** Reported in many contexts, especially routine settings
c) The design of effective models of care for delivering maternal ART involves many more consideration than the degree of integration.	**Strong** Evidence that integration effects are mixed and are determined by many factors is found in many studies of all qualities	**Moderate** Reported in several contexts, especially relevant in settings with weak health systems
d) Maternal ART has been under-prioritized in ANC, PMTCT and HIV programs.	**Weak** Often reported but mostly second-order interpretations	**Moderate** Reported in several different contexts
**Theme 2: Service Delivery**		
a) Dropout from and delays in the maternal ART cascade are exacerbated by a range of communication and coordination problems, including scheduling difficulties, poor follow-up and tracing of patients, weak information systems, and failure to keep up with rapidly changing treatment protocols and referral procedures.	**Strong** Detailed reporting in multiple well-designed studies	**Strong** Reported in many contexts, especially routine settings
b) Dropout from and delays in the maternal ART cascade are driven by problems in delivering HIV services in the context of ANC programs, including poor access to HIV testing, poor pre-/post-test counseling, lack of POC CD4 testing, and lengthy, rigid or complicated treatment protocols that made caring for sick or late presenting women more difficult.	**Moderate** Some elements of the finding stronger than others (e.g. CD4 testing), not much detail provided but consistent as a theme	**Strong** Finding reflected in many routine settings with high prevalence and weak health systems
c) Dropout from and delays in the maternal ART cascade were worsened by weak training and supervision of healthcare workers in the areas of emotional support (both for themselves and their patients) as well as up-to-date information on treatment protocols, referral procedures, and the importance of maternal HIV care.	**Weak** Reported by a few studies, often second-order interpretation with little detail on context	**Moderate** Finding reflected in some routine settings with weak health systems
**Theme 3: Resource Constraints and Governance Challenges**		
a) System-wide resource constraints that inhibit the access of those using health services, including human resources shortages and turnover, long waiting times, supply shortages and supply chain problems, and user fees, can pose an even greater barrier to accessing ART for pregnant women.	**Moderate** Widely reported in studies with high and low risk of bias but little context given on reasons behind barriers	**Strong** Widely reported in routine settings with weak health systems
b) Governance challenges in the broader health system, including centralized resource allocation, poor performance management and weak and fragmented accountability mechanisms, inconsistent payment processes, and the ineffective management use of health information, can exacerbate health service delivery problems at the facility level, especially for the services aimed at HIV-infected pregnant and postpartum women which are already fragmented, inefficient and poorly coordinated.	**Weak** Addressed in only a few studies, mostly second order interpretation but in studies with low risk of bias	**Moderate** Reported in a few studies in routine settings, consistent with other known barriers but little context provided
**Theme 4: Patient/Health System Engagement**		
a) There are many different aspects of the relationship between healthcare workers and HIV-infected pregnant women that have an effect on initiation, retention and adherence to maternal ART, including nature of confidentiality within the relationship, HIV-related stigma, favoritism, unequal power relationships, and perceptions about the healthiness of pregnant women.	**Strong** Widely reported in studies of varying quality, both qualitative and quantitative, often with adequate detail on context	**Strong** Reported in many studies in a wide range of settings, including routine settings
b) The success of efforts to initiate and retain HIV-infected pregnant women in ANC and HIV care was shaped by the directness, intensity, frequency, and extension of provider engagement with women.	**Moderate** Reported in several high-quality studies but not much detail on context, often second-order interpretations	**Weak** Reported in several studies but effects of factors varied
**Theme 5: Interventions to Improve Maternal ART Outcomes**		
a) While there may be any number of quick wins for improving outcomes along the maternal ART cascade, effective interventions typically move beyond integrating discrete elements of service delivery, and instead, provide multi-pronged and multi-leveled interventions in the broader health system to support maternal ART initiation, retention and adherence.	**Strong** Several studies with low risk of bias and sufficient detail across several intervention categories confirmed the finding	**Strong** Reported in several studies in routine settings, sufficient detail available.

In many cases, studies included important contextual information about health systems barriers and enablers that helped to explain their findings or added their own new information. We included information from both the findings and discussion sections of the studies. Discussion-related data was considered to be of less reliability than that reported under findings.

**Figure 2:**
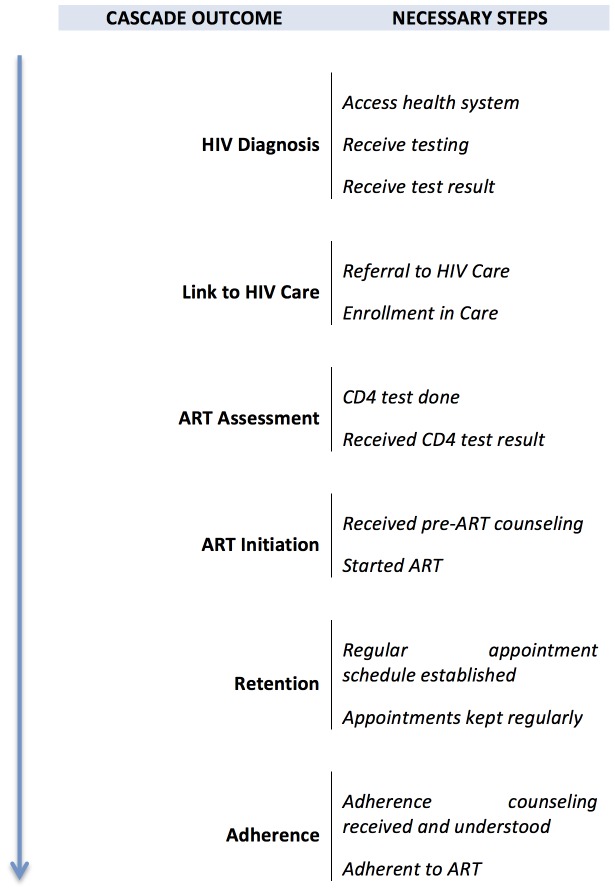
The Maternal ART Cascade.

Throughout the findings and discussion below, we also refer to the ‘maternal ART cascade’ to signify the multiple steps women must move through to initiate, be retained and/or remain adherent on ART. We distinguish the maternal ART cascade from the more commonly used ‘PMTCT cascade’ precisely because the steps that HIV-infected mothers must move through to initiate and remain adherent on ART for their own health are often broader and somewhat different than those maternal ART-related steps that are part of the PMTCT cascade (see Theme 1 below). Figure 2 captures some of the key steps women must move through in order to maintain successful treatment for their HIV infection, with the figure organized into key cascade outcomes in the movement along a path towards treatment and the component steps that are necessary to achieve each outcome.

Two further points about the cascade are important. First, each sub-step in the cascade is itself potentially quite complex. Even something as straightforward as having a CD4 test (one step in ART assessment) is multi-faceted. This step requires that providers remember the need for testing and offer testing to the client; that tests are available; that women accept the test; that the tests are available at the time and place they need to be done; that the client actually undergoes the test; and that the results are produced and made available by the clinic in a timely manner. Second, although the first step of this cascade begins with ‘Access to the Health System’, it is essential to remember that many women who are pregnant and HIV-infected do not access the health system early enough in their pregnancy to go through all the steps of the cascade. Issues related to this ‘total at-risk population’ are discussed in subsequent sections of this paper.

### Theme 1: Models of Care

#### Service integration and models of care

This theme explores questions about the design of various healthcare service delivery models and the extent to which they support ART uptake, retention and adherence for pregnant and postpartum women with HIV. The key issue of concern in most of the studies was the degree of integration between ART and maternal health services and the impact of different models of integrated care on outcomes. The studies reviewed described various models of care for HIV-infected pregnant women that represent differing relationships between adult ART and maternal health service integration. One study [42], for example, provided a useful typology of models for the integration of ART and ANC services, describing:

- ‘integrated’ models, where adult ART and ANC services were fully integrated,
- ‘proximal’ models, where adult ART and ANC services were separate but situated close to one another, usually in the same facility, and;
- ‘distal’ models, where adult ART and ANC services were located at different facilities, requiring both referral and travel for patients.

While many studies referred to the benefits of integrated models, only four of the studies compared two or more models of integration. Two of the studies found little difference in ART initiation between integrated, proximal and/or non-integrated models [42], [43]. Tsague et al. [43], however, found that women at the integrated ‘full-package’ sites were significantly more likely to be enrolled in HIV care and assessed for ART but no more likely than women at PMTCT-only ‘stand-alone’ sites to initiate ART. Two other studies found substantially improved initiation when services were integrated [53], [55]. The studies that found little difference in outcomes between the different models of care had small sample sizes and were conducted within existing services using routinely collected health data. Neither was powered nor designed to be a rigorous test of alternative models. The two studies that found differences were more rigorously designed and better powered. However, they were set within a more carefully supported and protected, and therefore, less typical context. Thus, findings from these studies are less generalizable and the models may be more difficult to scale up without substantial resources.

The researchers who found little evidence of a positive effect from integration were surprised by their findings and speculated that factors unrelated to integration could have been at work. Stinson et al. [42] point to chronic human resource shortages, rigid protocols, and lengthy drug readiness training requirements as reasons integrated clinics did not do significantly better than others. Tsague et al. [43] argue that broader improvements within the health system with respect to CD4 testing, healthcare worker training and strong patient follow-up may have reduced the observed differences between the ‘full-package’ and ‘stand-alone’ sites they investigated.

Both of these studies demonstrate that the design of effective models of care for delivering maternal ART requires the consideration of a multiple factors, in addition to integration (See Table 3, Key Review Finding 1a). In order to provide a richer understanding of the ways broader service design factors may affect ART outcomes, we have extracted aspects of service delivery that were identified frequently as important in the included studies. Rather than list all of these factors as a set of bullet points, we have reframed them as a set of questions designers or evaluators of health services would need to answer in order to develop an effective model of care, regardless of the degree of integration of services. These questions are presented in the first column of Table 4.

**Table 4:** Considerations for Maternal ART Models of Care.

ASPECT OF MODEL OF CARE	IMPLICATIONS FOR MATERNAL ART	CROSS-CUTTING ISSUES		
What kind of HIV testing is done, where is it done, and by whom?	Opt out is better for uptake. Lay counselors are more approachable but nurses provide more continuity. There is better continuity if HIV services are part of ANC consultations.	**RESOURCE CONSTRAINTS**	**OPPORTUNITY COSTS**	**POLICY OR POLITICAL BARRIERS TO SYSTEMS CHANGE**
Where is ART assessment and initiation done?	Referral and assessment is better in integrated services, but it is not clear if initiation is better.			
Who is allowed to initiate ART?	The more cadres that can initiate ART, the more flexible and responsive initiation can be.			
How are lab services integrated with ART assessment and initiation services?	Point of Care CD4 testing can speed initiation.			
What mechanisms (record-keeping, appointment systems, peer escorts, etc.) are used to ensure linkage across different services?	More active linkages between services and follow up/tracing (follow up by CHWs) result in fewer drop-outs.			
How are women transferred into and out of adult ART services before and after pregnancy?	For women already on ART, management of ART while pregnant needs to be coordinated with PMTCT program. For these women, and for women not on ART before pregnancy, how they are transferred back to general adult ART services can impact retention and adherence.			
How are the general adult ART services organized?	More decentralized ART services, in general, make linkages between ART and ANC services easier.			
What is the intended pacing of movement through the maternal ART cascade?	Drug readiness, training, PMTCT protocols, and other assumptions built into models of care can greatly increase or decrease the pace of movement through the maternal ART cascade, and thus, opportunities for drop-out.			
Are CHWs, peer mentors or support groups part of the model of care?	These cadres/interventions can provide better psychosocial support for women throughout the cascade, as well as contribute to coordination of and linking across services.			

The second column in Table 4 summarizes some of the implications of particular design choices for maternal ART, as described in the included studies. The three issues that comprise the third, fourth and fifth columns of the table relate to cross-cutting factors identified in the studies that constrain the kinds of design choices that are possible. Resource constraints can make some choices less tenable, opportunity costs may make some choices less desirable, and policy and political barriers to change may make some choices less feasible.

#### Inconsistent attention to maternal ART

One key recurring theme in several of the included studies was a loss of focus on, or prioritization of, maternal ART within ANC, PMTCT and HIV programs (See Table 3, Key Review Finding 1d). There were frequent references to the perception that, in the process of prioritizing the prevention of vertical transmission, management of the mother's HIV infection was relatively neglected, often to the longer-term detriment of the health of both mother and child.

One study [46] found that adult ART services sometimes refused to enroll pregnant women with HIV on the assumption that they were healthy enough to wait for assessment until after delivery. One clinic in that same study even reported that HIV nurses would turn pregnant women away on the mistaken assumption that they had already received the appropriate HIV treatment and care—either ART for their own health or as prophylaxis—in the PMTCT program. These two examples reflect the findings of a number of other studies [18], [30], [47], [53].

The unique needs of HIV-infected pregnant and postpartum women also appeared not to be well-accommodated by HIV and maternal health services. Several studies found that postpartum women were particularly difficult to retain in care, given the demands they faced caring for a newborn, given that many maternal health sites offered few postpartum services, and given that many ANC sites had weak referral systems to adult ART services. Antenatal services were generally described as disconnected from non-pregnancy related services such as general pediatric care or general HIV care, and there was a high rate of dropout both from the health system more generally, and from ART specifically around the intrapartum period. For their part, adult HIV services also did not seem to take into account the many social, physical and emotional challenges that mothers faced during the postpartum period [16], [19], [27], [30], [31], [36].

Similarly, two studies reported that women, whose CD4 counts were too high to beeligible for lifelong ART at the time of testing or who refused lifelong ART during pregnancy, had a very high postpartum dropout rate and therefore merited special attention. Ideally, these women would have been retained in HIV care to support their health, address opportunistic infections, and be regularly re-assessed for ART eligibility [19], [20].

Finally, most of the models of care that were reviewed neglected, or actively excluded, male partners of pregnant or postpartum women from participating in maternal health services, especially ANC and HIV care. It is often difficult to include male partners in HIV testing and treatment sites for women, especially in the antenatal context of HIV care for pregnant women [18], [22], [23], [26], [32], [36]. HIV services, thus, often failed to adequately retain or include a range of relevant sub-groups in care—including postpartum women, ART-ineligible and ART-declining women, and their male partners. (See Table 3, Key Review Finding 1c)

#### Persistent service gaps and maternal ART cascade dropout

Even when new models of care were carefully designed to support pregnant or postpartum women's access to ART and overcome typical access barriers, such as those related to vertical services and physical distance, long wait times, and fears about loss of confidentiality, dropout along the maternal ART cascade often remained a persistent problem (see Table 3, Key Review Finding 1b).

Several studies described services that were located next to each other (proximal models of integration) but remained poorly coordinated due to barriers such as communication and failures in coordination, or bottlenecks related to the delivery of lab results. In some cases, poor service delivery was the result of integrating some aspects of care (for example, including HIV testing as part of ANC care) without attending to broader system weaknesses (such as the poor availability of test kits) [5], [17], [19], [42], [43], [53].

Task shifting is an approach to addressing the shortage in human resources. In several studies, rigid task allocation and treatment protocols that prevented nurses from initiating ART were identified as significant barriers to access [42]. However, task-shifting can also present challenges when not carefully considered and supported. One study also found that task shifting could contribute to more fragmented care by shifting HIV testing to lay counselors, thereby complicating the ANC care pathway between nurse and patient, and increasing workloads [57].

Breaches in confidentiality that resulted from fragmented services emerged as a barrier to access as well. In two studies, separate waiting rooms and the use of different types of medical forms for those accessing HIV care led to fears around the identification of those receiving HIV care [16], [18]. Other studies found that outreach efforts intended to improve patient follow-up (such as home visits or food baskets for new mothers) threatened confidentiality, and thus, retention in care [18].

There were several studies that discussed models of integration that were able to improve retention throughout the maternal ART cascade. Killam et al. and Ramers et al. both reported that integration of ART services into ANC roughly doubled the number of eligible women initiating ART [53], [55]. Weigel et al. described a multi-faceted intervention to improve linkages between HIV and ANC services that similarly improved ART initiation and retention [47]. This intervention included provider-initiated testing and counseling, queue prioritization, same-day blood collection for CD4 testing, and ‘fast-tracked’ ART initiation.

No one model of care included in this review was able to fully address all of the barriers to initiation, retention and adherence. In most of the studies, health services failed to adequately enable and support ART initiation and retention for the majority of pregnant or postpartum women. The more effective models ensured coordination among various components of the system and focused specifically on overcoming the challenges associated with treating HIV-infected pregnant and postpartum women, and in particular, the period of transition between pregnancy and post-partum periods for individual women.

### Theme 2: Service Delivery

This theme addresses the day-to-day management and operation of service delivery. The included studies described a wide range of service delivery factors that affected initiation, adherence, and retention. Most of the reported issues involved poor communication and coordination, but there were also problems with the quality of clinical practices and significant gaps in training and supervision. While some of these problems reflected weaknesses in the broader health system, many of them were either specific to or intensified by the unique challenges of providing ANC and PMTCT services to this population.

#### Communication and coordination problems

A wide range of the included studies reported that dropouts and delays in the maternal ART cascade were often the result of communication and coordination problems, including scheduling difficulties, poor follow-up and tracking of patients, weak information systems, and failure to effectively communicate rapidly changing treatment protocols and referral procedures throughout the health system (See Table 3, Key Review Finding 2a).

Scheduling difficulties of all kinds appeared in the studies and were driven by a variety of factors. Problems included overbooking, cancelled bookings, failure to book on time, and services that were available only on a very limited schedule [27], [41], [44]. Some of the challenges posed by these scheduling issues were compounded by demand-side factors such as late presentation for ANC services or missing scheduled ANC visits that then threw off the often rigidly scheduled ANC service schedules (detailed in the next section) [19], [35], [45]. Other challenges were related to perceptions among healthcare workers that pregnant women were relatively healthy compared to other patients and could be among the first patients to be sent home when the clinic was overcrowded [18].

When women did not show up for appointments, there were often no systems in place to track and follow up with them to ensure they continued to receive care. Women's own perceptions that they are healthy while pregnant and do not need care, and their tendency to move to new locations during or after pregnancy to access family support [17], [24] compounded the challenge associated with enabling their continued access to services. These demand-side factors highlight weaknesses in the health system, including its general orientation towards reactive engagement with patients (i.e., waiting for them to present) and lack of coordination across services and facilities. These weaknesses translated into few mechanisms for either tracking women who dropped out of ANC, PMTCT, postpartum services, or HIV care, or keeping track of unplanned or unsuccessful referrals across facilities. Only a couple of studies identified processes for quickly identifying and following up on missed appointments (see Theme 5 and the Supporting Information for more information on effective interventions) [17], [47].

The problems associated with scheduling, follow-up and referral are largely generic to healthcare provision for pregnant women in general, although the stigma associated with HIV adds a layer of complexity. The reviewed studies also identified challenges for communication and coordination which were specific to PMTCT and ART programs. In particular, rapidly changing PMTCT and ART protocols often outpaced the ability of the health system to appropriately adapt. When new treatment protocols or changes in models of care were introduced, they were often not fully planned out at the level of day-to-day service provision and critical issues such as workload distribution, task allocation, patient flow, and record-keeping and appointment keeping systems were not adjusted [29].

The slow and uneven diffusion of information about these rapid changes in protocol through the health system was also noted in several studies. Lack of awareness on the part of nurses about the most recent changes in ART protocols, clinical practice guidelines, and/or the organization of service delivery—in particular, referral pathways beyond their facility—were identified in several studies as barriers to effective clinical care and patient retention [22], [24], [27], [46].

In several contexts, weak systems for disseminating information about new guidelines, policies and practices were identified as an important underlying cause of many of these communication and coordination challenges [31], [33], [37], [41]. This weakness reflects broader health system challenges.

#### Clinical service delivery issues

Dropout from and delays in progression along the maternal ART cascade were also driven by problems in delivering HIV services in the context of ANC programs, including poor access to and quality of HIV testing, lack of quick CD4 testing, and lengthy, rigid or complicated treatment protocols that made caring for sick or late presenting women more difficult (See Table 3, Key Review Finding 2b).

Several studies reported that rigid or inappropriate treatment protocols could make it difficult for ANC, PMTCT or HIV services to accommodate the needs of pregnant women or adjust to their early or late presentation to the health service. One study [27], for example, found women who presented early at ANC, in their first trimester, had a higher risk of not initiating ART than those who presented later since the protocol at the time (in 2008) only allowed initiation of prophylaxis at 28 weeks. Most other studies, however, reported a contrasting problem—PMTCT and ART treatment protocols were multi-step and organized around an assumption that women would present at the ‘right time’ for ANC services [19], [26], [35]. Consequently women who presented late had difficulty moving through all of the ART steps—diagnosis, assessment, preparation, initiation and maintenance—before delivery.

A number of studies reported that poor access to HIV testing and poor quality of pre- and post-test counseling also were significant barriers to women initiating ART. If testing is not easily available to women during the ANC visits or if counseling is of poor quality, women can be deterred from testing or pursuing HIV care once diagnosed with HIV, missing important linkage opportunities. In some situations, these testing barriers reflected more generic problems with overall HIV testing services, but in other cases, there were problems with access to and quality of HIV testing specific to ANC settings [31], [41], [42]. The absence of high-quality couples testing and counseling was also seen as a significant barrier, especially to male partner involvement in ANC and general HIV care [26], [39]. A few studies reported that high-quality HIV testing, prompted or opt-out HIV testing, and inclusive couples counseling were significant enablers of ART uptake and male involvement in ANC and PMTCT services [25], [41], [48].

Finally, the next step along the maternal ART cascade—assessment—proved to be as difficult as the step discussed above. Problems related to CD4 testing were reported as the next critical barrier in movement along the cascade for women who were referred to and successfully entered HIV care for assessment [29], [33], [41], [44], [46]. In settings where point-of-care CD4 testing was not available, blood samples had to be sent to offsite laboratories. Both the time required for conducting tests and returning results and frequently delayed or missing test results were critical barriers to assessment, and thus initiating ART.

#### Training and supervision

Poor training and weak supervision were the third area identified as contributing to low quality service delivery and drop out and delay along the maternal ART cascade. Providers often did not have the requisite skills or knowledge of current treatment protocols or referral procedures for providing maternity care to HIV-infected women, and were unprepared to provide emotional support to their clients. Insufficient supervision also meant that the providers themselves did not receive emotional and other requisite support (See Table 3, Key Review Finding 2c).

Other aspects of PMTCT and HIV care for pregnant women posed additional challenges to initiation, retention and adherence [22], [24], [27], [33], [38]. Rapidly changing PMTCT and ART protocols required frequent retraining and supportive supervision. Nurses who were trained before the widespread availability of ART or PMTCT were at a disadvantage but even more recently educated healthcare workers sometimes struggled to maintain up-to-date knowledge about treatment protocols and other aspects of clinical care. Finally, reflecting a broader lack of focus on the health needs of HIV-infected mothers, some antenatal healthcare workers were reported as having insufficient awareness of the importance of maternal ART, leading to a failure to encourage and follow-up on HIV care for these women [30].

A few studies also reported on weak training and supervision for nurses with respect to stress management and emotional support, both for their patients and themselves [24], [41], [45]. This gap was seen in the lack of preparation for responding to the special emotional needs of pregnant women who often feel they are healthy, are excited about their pregnancy, and yet, at the same time, feel very concerned about their HIV status. Healthcare workers' often stigmatizing attitudes towards pregnant women with HIV compound the challenge of providing emotional support for these women. A lack of training and supervision, likewise, left healthcare workers poorly prepared to manage their own stress and feelings of cognitive dissonance about the co-presence of pregnancy and HIV.

### Theme 3: Resource Constraints and Governance Challenges

The studies also reported a range of issues associated with weaknesses in the broader health system, weaknesses that were often the result of resource constraints and governance challenges.

#### Resource constraints

System-wide resource constraints such as human resources shortages and turnover, supply shortages, and poor supply chain infrastructure also could be barriers to accessing ART for pregnant women (See Table 3, Key Review Finding 3a). Human resource shortages and high staff turnover were reported to have substantial effects on maternal ART initiation, retention and adherence, largely because these factors prevented effective continuity of care [18], [24], [31], [41]. A number of studies also reported supply shortages and supply chain problems that affected maternal ART outcomes. Drug stock-outs or the absence of sufficient numbers of HIV testing kits led to drop-out at several points along the maternal ART cascade [39], [41], [54].

Some of the most common complaints from pregnant and postpartum women were related to features of the health system that can also be traced to these wider resource constraints. For example, long waiting times was the most frequently cited barrier to accessing HIV care in the included studies [16], [18], [23], [29], [33], [38], [48]. Long waiting times were at least partly the result of poor scheduling and management of patient flow in most settings, and were also the result of deep and persistent human resource shortages.

Long waiting times represented a particularly important barrier for HIV-infected pregnant women for three reasons. First, some studies reported that pregnant women were more likely to feel that their pregnancy made them less able to wait for long periods of time. Second, by contrast, many women, along with their families and healthcare workers, perceived pregnancy to be a time of health rather than illness, making long waiting times seem all the more unnecessary. Finally, given the widespread association between pregnancy and health, some women feared that spending a long time at the clinic would raise questions among family and community members about whether they were seeking HIV care. The fragmentation of ANC and HIV services exacerbated this problem by compounding the total length of waiting time.

#### Health system governance

In addition to the effects of resource constraints, some of the studies also reported a range of broader health system governance challenges. The challenges included overly centralized resource allocation, poor performance management, weak and fragmented accountability mechanisms, inconsistent payment processes, and the ineffective management use of health information. These challenges exacerbated health service delivery problems at the facility level, especially the services for HIV-infected pregnant and postpartum women, which often are already fragmented, inefficient and poorly coordinated (See Table 3, Key Review Finding 3b).

Centralized resource allocation, for example, can act as a barrier to effective maternal ART services at the facility level by taking local-level spending flexibility away from lower levels of the health system [41]. Similarly, poor performance management processes, and weak and fragmented lines of accountability within and between services (e.g., between ANC and HIV services) sometimes made it difficult to identify problems in ART and ANC service delivery and to respond appropriately and effectively [23], [27], [41]. Even simple breakdowns in management systems, such as inconsistent payments to healthcare workers, or more frequently, to lay counselors conducting HIV counseling and testing, could result in significant reductions in morale and quality of care [41].

Another management weakness, linked to the weakness of health information systems noted above, was that management largely did not use health information for making decisions, in allocating resources, or in accountability. Even when some form of routine data was available to health managers, there was often a failure to make use of this information for management purposes. This lack of data use was especially true with information related to maternal ART, reflecting again, the earlier key review finding about insufficient focus on maternal ART [41], [47], [48].

### Theme 4: Patient-Health System Engagement

This theme considers the engagements between HIV-infected pregnant and postpartum women, and the range of providers in health facilities, from clinicians to community health workers and others. The ‘health system’ framework and the term ‘engagement’ are used here to signal a broader range of engagements than ‘patient-provider interaction’ alone. All forms of interpersonal practice, perception and relationships that more broadly impact women and their degree of ‘engagement’ with the health system are discussed below.

#### Complexity of the patient-provider relationship

In several studies, the relationships between the women and the healthcare workers with whom they interacted were a rich source of commentary and critique. While many studies reported generically that ‘negative attitudes’ or disrespectful providers were a barrier to care, there were also other important aspects of the relationship between healthcare workers and HIV-infected pregnant and postpartum women that had an effect on initiation and retention to maternal ART. These included the nature of confidentiality within the relationship, HIV-related stigma, favoritism, unequal power relationships, and perceptions about the healthiness of pregnant women (See Table 3, Key Review Finding 4a).

Negative and stigmatizing attitudes of nurses and doctors were a common complaint of patients in many settings, regardless of the clinical service. Women in these studies complained about the general negative attitudes of nurses and doctors in the public health service, as well as stigmatizing attitudes related specifically to pregnancy, sexual behavior and HIV [18], [22], [23], [26], [41], [45], [54]. Besides making women feel judged and unwelcome, HIV-related stigma among healthcare workers was also frequently perceived by women to pose a direct threat, i.e., the possibility of confidentiality being breached. There were widespread fears among women that nurses, in particular, would either intentionally or unintentionally disclose their HIV status to others in the clinic or community out of either malice or reckless disregard [39], [45].

Women in some studies also reported concern about favoritism or nepotism among healthcare workers at their local clinic [16], [18], [23]. Some indicated a preference for going to facilities outside of their community, not only for the confidentiality protections this might afford but also as a way to avoid social discrimination within their local facility [18], [45]. This discrimination could be family-based, religious or ethnic in nature.

Staff attitudes towards pregnant women were also shaped by their perception of pregnancy as a state of positive health and the behavior of pregnant women. Several of the studies reported a more general perception that pregnant women were essentially healthy and that when the clinic was full or resources were tight, they could receive less attention or be asked to return on another day [18], [24], [46].

In general, the studies describe a relationship between patients and providers that is persistently highly unequal in terms of social power. This was less true of engagements with community health workers, with whom women generally had positive relationships [45]. Doctor-patient relationships were highly unequal, but in most of the settings assessed, doctors were not a frequent presence. It was the nurse-patient relationship that received the most attention and concern in the reviewed studies, and was most frequently described as both asymmetrical and disempowering.

A few studies reported on positive relationships between patients and providers. These generally found that positive relationships resulted in dramatic differences in perceptions about the quality of care and patient satisfaction, as well as uptake and retention in care. Several studies, for example, reported that provider characteristics were critical factors in HIV testing uptake, and could be far more important than other health systems barriers, such as long waiting times for tests or un-integrated services [18], [25], [41]. These findings highlight the importance of respectful care throughout all aspects of reproductive, maternal and HIV service delivery.

#### Characteristics of effective forms of engagement

Besides the direct, interpersonal relationship between patient and provider, there were a number of other important forms of engagement described in the included studies. The more effectively health services kept women engaged with the health system, the more likely women were to initiate and be retained in care. There were several ways in which health systems kept HIV-infected pregnant women engaged in maternal health and HIV care. Factors that contributed to success of these approaches included their directness, intensity, frequency, and extension of providers' engagement with women beyond the health facility (See Table 3, Key Review Finding 4b).

The power of more direct, intensive and frequent health system engagement was a theme of several studies that reported on women's need for more health information and health counseling opportunities at regular intervals. These studies argued that many women lacked opportunities for significant one-on-one interaction with a healthcare worker and that when these opportunities were available, they were a critical enabler of positive maternal ART outcomes [17], [23], [33], [34], [44]. Women were seeking health information, as well as counseling with respect to health decision-making and the emotional dimensions of their HIV diagnosis [23], [24], [41], [46]. They were often uncomfortable seeking this advice in group contexts, in public waiting rooms, semi-public consultation rooms, or with providers to whom they had little prior relationship.

Repeat visits to health facilities for ANC or HIV care were described as important predictors of initiation, retention and adherence to ART [19], [22], [24], [28], [46]. This could be due to the fact that repeat visits provide more opportunity for the kinds of direct and intensive engagement described above. The direction of causality is not necessarily clear, however, since women who attend ANC services regularly may already be more likely to initiate and remain adherent to ART.

Outreach efforts like home visits or SMS reminders, when not a threat to disclosure, were described as enablers of better outcomes if they resulted in more consistent or direct contact with women.

Several of the studies reported on a positive association between accessing ANC care or having a facility birth and better maternal ART outcomes, at a more general level [22], [26], [28], [31], [39]. This was a strong and consistent association. However, there was insufficient detail as to the direction of this effect. Again, it is not clear whether facility birth somehow produces the conditions for better, ongoing HIV care, or whether those who have facility births are more likely to initiate or adhere to ART. Nonetheless, there was a consistent pattern that poor retention during ANC was associated with poorer maternal ART outcomes, and ART initiation before delivery was associated with better retention and adherence during the postpartum period.

### Theme 5: Interventions to Improve Maternal ART Outcomes

This theme reviews findings related to interventions for improving outcomes related to maternal ART. The majority of the studies did not report on specific interventions. Some evaluated the outcomes of new treatment protocols or models of care but did not formally compare these outcomes to other or previous protocols or models.

Twelve studies (29 percent), however, assessed the effectiveness of specific interventions to improve initiation, retention and/or adherence to ART among HIV-infected pregnant or postpartum women [21], [35], [37], [38], [42], [43], [47], [48], [52], [53], [55], [56]. Four of these studies assessed newer service delivery models [42], [43], [53], [55] and one involved the Option B+ for PMTCT treatment protocol. [48]. Four of the studies described more discrete interventions: an SMS support group, two ‘fast-tracking’ initiatives to increase the speed of ART initiation, and a pilot test of simultaneous triple point-of-care testing for HIV, syphilis and hepatitis B [21], [35], [37], [38]. Three studies described much more comprehensive, multi-layered efforts to intervene across the ART cascade and within different aspects of the health system [47], [52], [56]. A summary of these interventions along with the qualitative and quantitative mechanisms and effects reported by the studies is available in the Supporting Information.

The design of interventions, the intervention settings, the length of study follow-up, and the ability of these studies to validly detect differences between different models of care/interventions varied widely. Generalizing about which interventions for maternal ART might be most effective is, therefore, not really possible with this set of data. However, one broad theme that can be derived from these findings is that while there may be some potential ‘quick wins’ in improving outcomes along the maternal ART cascade, the most effective interventions address more than one area of concern. These provide multi-pronged and multi-leveled interventions along the maternal ART cascade and within the broader health system to support maternal ART initiation, retention and adherence (See Table 3, Key Review Finding 5a).

For example, the integrated ANC and ART services for mothers described by Wiegel et al. [43] showed dramatic improvements in maternal ART outcomes. It is important to note, however, that in addition to service integration, this program involved wide-ranging interventions throughout maternal health and ART services. The effect of the introduction of Option B+ in Malawi on maternal ART initiation was similarly dramatic [48], [50], leading to a 748 percent increase in the number of initiations over a one-year period. Here, too, however, the intervention involved far more than a change in protocol (i.e. removing the requirements for CD4 staging and starting all pregnant women on ART when they test positive for HIV). ANC and ART services were thoroughly decentralized and integrated, health information systems strengthened, task shifting implemented, healthcare workers were trained in the new guidelines, drug regimens for pregnant women were changed, and there was intensive supervision and support from a national task team.

More narrow, targeted efforts to reduce delays in movement along the maternal ART cascade, such as ‘fast-tracking’ of ART [35], [37], also significantly improved ART uptake. Evidence about how sustainable or widespread the impact of these more discrete interventions may be, is not yet available.

## Discussion

### Maternal ART in the Changing HIV Landscape

Global efforts to scale-up universal access to HIV treatment and care and intensify PMTCT programs continue to gain pace. Ambitious goals of 15 million people using ART and reducing the number of new infections in children by 90 percent by the year 2015 are gaining momentum, and there have been some resounding successes [58]. HIV testing and ART initiation rates are improving around the world and many programs in resource-constrained settings are reporting retention and adherence rates that match or surpass those in developed settings [7], [59].

There are also some emerging challenges as HIV becomes a chronic disease and the delivery of lifelong ART becomes a central component of the ongoing work of health systems. The financial sustainability of and political commitment to HIV and AIDS programs, the persistent shortage in human resources for health, and the challenges of long-term treatment adherence are of increasing concern in this new era of massive public ART programs [60]. These challenges will only intensify as global policies moves towards universal treatment.

The program outcomes reported by the studies included in this review argue for additional concern when it comes to maternal ART. There were significant drop-outs throughout the maternal ART cascade and the numbers of women who ended up receiving treatment compared to those who needed it (the ‘treatment coverage gap’) was often worse, and sometimes much worse, than outcomes reported in general adult ART programs. It appears that ensuring pregnant and postpartum women are enrolled, retained and adherent on ART for their own health poses unique challenges for health systems.

Many of the health system barriers to and enablers of maternal ART that we identified in this review will be familiar to those working in adult ART contexts. Problems with communication and coordination among healthcare workers and services, stigmatizing healthcare worker attitudes, supply chains, and poor health information systems were common weaknesses of many health systems. Addressing these problems is not only a priority for the health of HIV-infected pregnant and postpartum women but is also essential for ensuring the health of all segments of the population. But these factors must receive particular attention if the global goals of scaling up ART and PMTCT programs are to be successfully implemented.

This review identified a number of health system-related factors that were related specifically to the experiences, needs and vulnerabilities of pregnant and postpartum women which are of relevance for program managers and policymakers working to reduce HIV-related maternal deaths. Table 5 provides a summary of the health system factors of relevance to caring for pregnant and postpartum women.

**Table 5:** Pregnancy-Related Health Systems Factors.

PREGNANCY-RELATED HEALTH SYSTEMS BARRIERS AND ENABLERS
Challenge of coordinating HIV care for women with their movement into ANC, through the delivery and post-partum phases of care, and back into general adult primary care
Loss of focus on pregnant women's health needs in the context of PMTCT programs' focus on preventing vertical transmission
The particular blind spot of the postpartum period, when women are transitioning out of ANC and PMTCT care but not are effectively linked to ongoing primary or HIV care
Health services that are not set up to respond to the unique health and social needs of pregnant women (e.g. not sitting and waiting all day to be seen, not being viewed (by selves and others) as ‘sick enough’ to warrant focused attention, need for movement across facilities during and after pregnancy to access social support, difficulty in coordinating both ANC and HIV care visits, if separate)
Poor knowledge and training among healthcare workers about the importance of maternal ART, maternal ART protocols and maternal ART referral procedures
Time-bound nature of pregnancy not sufficiently accounted for in some ART protocols that required lengthy assessment and drug readiness training before initiation

While the studies we reviewed provided rich detail on the kinds of health system barriers and enablers to ART that existed for pregnant and postpartum women, we found less evidence about the kinds of interventions that might prove successful in addressing health system barriers [61]. There were several interventions that showed very promising results; these tended to involve intensive, comprehensive, and well-supported interventions. The question of whether these interventions can be scaled and still produce similar results remains open and urgent. Other studies that tested simpler or shorter-term interventions in routine settings reported less dramatic results. Another consideration when interpreting the findings about intervention effectiveness is that most of the available evidence comes from studies that were not designed to carefully compare different interventions. They may not, therefore, on their own provide the kind of detailed and reliable evidence needed to guide maternal ART programs and policies.

Some studies that did not fit the review inclusion criteria of providing specific findings on health system barriers and enablers to ART nonetheless described promising interventions for maternal ART programs. One was a community health worker (CHW) program in Malawi that provided more integrated care for and sustained contact with HIV-infected pregnant and postpartum women and reported a number of improved maternal ART and PMTCT outcomes [62]. Another similar intervention using CHWs in South Africa is also showing promising results [63]. Whether or not these CHW initiatives for providing support to pregnant women during testing, ART assessment and initiation, and retention and adherence offer a potential solution to some of the health system barriers and enablers discussed in this paper is an open question. An important commonality of these interventions, with the successful ones included in this review is their systems-oriented approach to health system problems and their focus on intervening along several points of the maternal ART cascade. This also underscores the importance of careful evaluation to identify where along the cascade problems exist and tailoring interventions to address the specific problems identified.

‘Option B+’, a PMTCT treatment protocol that makes all HIV-infected pregnant women eligible for ART regardless of CD4 count, is increasingly being adopted by many countries. This protocol removes a critical barrier to initiation, the need for CD4 staging, and allows women to move as fast as possible from HIV diagnosis to treatment initiation, thus, increasing their health and survival outcomes. Only one study included in this review directly assessed Option B+. However, it was a very brief and early report on program outcomes. It described a fairly comprehensive national intervention in Malawi that showed remarkable success in initiating much greater numbers of pregnant HIV-infected women onto ART.

The other evidence reviewed, while indirect with respect to Option B+, provides both notes of support and caution for this promising approach. Studies included in this review suggest that Option B+ may be successful because of the ways it addresses the critical drop-outs along the several steps that typically separate HIV diagnosis and ART initiation. By simplifying the PTMCT protocols related to CD4 staging, healthcare workers would no longer be required to wait to receive CD4 results before initiating ART.

Option B+, however, does not lessen the need for thinking carefully about 1) the organization of care with regard to the integration of HIV and ANC care; 2) how pregnant women's movement across and between these services can best be facilitated to ensure they are not lost to follow up; 3) how to encourage women to accept treatment and manage those who may not be ready to initiate soon after diagnosis; and 4) how to ensure they are retained in care and adherent to ART throughout their pregnancies and postpartum.

We cannot predict how successful Option B+ programs will be in addressing all of these challenges. It is apparent that pregnant women will continue to present unique challenges for maternal health, PMTCT and HIV services. It is also clear that focused attention is warranted to ensure that their enrollment and maintenance in HIV care is carefully and effectively integrated throughout the complicated and vulnerable period of pregnancy and postpartum. Option B+ will resolve some of the key obstacles to initiating HIV-infected pregnant women onto ART, which are a critical concern given the time-bound nature of pregnancy. Nonetheless, many of the broader health system challenges and concerns around maternal ART programs identified by this review will remain relevant and will need to be answered by local policymakers and program managers in ways appropriate to local contexts and constraints.

### Review Limitations and Research Gaps

#### Limitations of the review design and scope

The strengths of this review's design included its inclusive search strategy that ensured wide coverage and the dual data extraction and iterative analysis. There are several important limitations to note. Time constraints prevented a more thorough and systematic search of the gray literature, a body of evidence that may have had more to offer with respect to Option B+ and other current maternal ART program experiences and interventions. Time and resources available also limited the degree of outside, expert review that was possible and the languages that could be covered.

While time may have also restricted the degree to which we were able to refine our analysis and further transform the data from the included studies, it is not clear we could have done much more beyond thematic and cross-case comparative analysis, given the diversity of the evidence base with respect to settings, ART/PMTCT program types and protocols, and study questions and designs. The diversity of this evidence is a strength in terms of the richness of data, but it was hard to identify widely generalizable patterns or compare across studies with respect to specific program outcomes, health system factors, or study quality.

While not a design limitation per se, the scope of the review centered on studies reporting health system factors specifically related to maternal ART. Much of the adult ART and PMTCT-related literature on health system factors and potential interventions would have yielded valuable lessons that might have been transferable to the problem that was the focus of this review. This, however, was beyond the scope of the review.

#### Gaps in the evidence base

Our review noted a number of important gaps in the evidence base related to the health system and maternal ART. Perhaps the most important gap is the women who are not represented in the data—those who did not make it to ANC or HIV testing in the first place, or who dropped out along the maternal ART cascade. Given the health system-centric nature of much of the research, we know much more about who stayed in care and why than about those who never attended ANC or who dropped out.

A related concern is the tendency in the studies reviewed was to measure initiation or retention rates using a denominator drawn from the closely preceding step in the maternal ART cascade. For example, an initiation rate might assess the number of people who started treatment at a clinic against those who were assessed by the clinic as eligible for treatment. This figure, while meaningful as a measure of effectiveness of a particular clinic, obscures other potentially more important measures of health system function. In these studies, the number of people who started treatment might be compared against the total number of ART-eligible patients who were successfully referred to assessment, or against the total number of ART-eligible patients who received an HIV test, or even against the total population in need, i.e. all of the HIV-infected and ART-eligible pregnant women in a catchment area (many of whom never access the health system). Alternative comparisons like these would highlight the scale and impact of cumulative dropout along the maternal ART cascade. Measures of dropout with a narrower frame of reference make it harder to think broadly about the challenge of supporting pregnant women all the way through the maternal ART cascade. Deciding on the most relevant basis for comparison in health statistics is not only a challenge for research but an important one for policy making, program planning, service delivery and management since the choices made shape the factors that are more and less visible.

Ensuring that women initiate ART and remain in care is difficult, and the problem of long-term adherence is equally important and complex. The review identified several weaknesses in the evidence base that are of relevance to these challenges. The first is the absence of evidence about long term adherence. Most of the studies included in this review assessed initiation and a few assessed retention in care (the easiest longitudinal variable to assess using routine health data in clinical epidemiology studies). We found very little on adherence. This likely reflects the broader lack of attention to maternal ART outcomes that have been discussed above.

The second adherence-related gap is the lack of consistent, and thus, comparable measures of adherence across studies. In Table 2, we reviewed the measures of adherence used in the included studies. As with the question of which denominator to use when assessing drop-out, unless there are measures of adherence that are as valid, reliable and consistent as possible, it will be very difficult for future researchers to assess this critical long-term program outcome.

We have already noted the relative lack of well-designed, prospective studies of maternal ART outcomes and comparisons of models of care or other interventions in routine settings. There is also an absence of data from outside of Southern and Eastern Africa.

Finally, we were struck by how few studies explicitly modeled the dimension of time as it related to movement through the maternal ART cascade [34]. Most of the studies that assessed factors related to drop-out mentioned timeliness (moving quickly through the cascade) and timing (aligning phases of the pregnancy, clinic visit schedules, and ART protocols) as critical considerations, given the relatively short, time-defined nature of pregnancy and the complicated steps often required to move women through both ANC and HIV care. However, very little empirical evidence was collected or analyzed about timeliness or timing as discrete variables. In the absence of such information, it is not only difficult to establish if, to what degree, and to what effect particular interventions may speed up womens' movement through the cascade. It is also difficult to address potentially more complex questions such as the optimal timing of movement through the cascade when consider in relation to other factors that might recommend less rapid initiation, such as poor treatment readiness or the presence of co-morbidities in pregnant women [34], [64], [65].

This issue is critical since approaches like Option B+ are often mistakenly described as interventions that inevitably reduce the time it will take women to move through the cascade. The simplification of PMTCT protocols and subsequent time required to initiate treatment are often inter-related. However, it is critical to remember that even with Option B+ in place, there could be any number of critical barriers to initiation that significantly delay ART initiation. Conversely, Myer et al. [35] showed that even within the framework of Option B (ART initiation at CD4 <350), programs could find ways to ‘fast-track’ eligible women and enable significantly more of them to initiate ART the same day they were diagnosed. It, thus, seems important to give added attention to defining and collecting data on timeliness and timing as discrete variables in studies of maternal ART processes and outcomes.

### Conclusions and Recommendations

Policy and program responses to our review findings will inevitably need to be highly context specific. We have, therefore, developed over-arching conclusions and recommendations to guide this process. Table 6 presents the key conclusions from this review in the left column and outlines a series of recommendations in the right column that can be used to guide policymakers and program managers in applying the findings within their local settings.

**Table 6:** Recommendations.

Focal Area	Recommendation
**Strengthening the Focus on HIV-infected Pregnant Women**	Policymakers and program managers should re-evaluate maternal ART services using the maternal ART cascade and pathways through maternal ART care as organizing frameworks. This re-evaluation should 1) systematically diagnose current bottlenecks and drop-out points in detail, 2) identify the health systems barriers that contribute most to these problems, 3) identify a set of interventions that could sustainably and effectively addresses these problems, and finally, 4) align these priority areas and intervention options with existing HIV and ANC/PMTCT services.
	Policymakers, program managers and researchers should focus on understanding and addressing delays in progression through the maternal ART cascade. Both monitoring and evaluation processes should include measures of time and its effects more explicitly.
	Attention should be renewed on the range of women who never make it to ANC or HIV care or who drop out along the cascade. More information on who they are, why they never access care or why they drop out needs to be collected. Interventions to recruit, retain *and* trace those lost to follow-up should be prioritized. Statistics documenting attrition should include a wider range of denominators when assessing program outcomes.
**Critical Interventions to Support ART for HIV-infected Pregnant Women**	Health management information systems for maternal ART need to be improved. This requires strengthening both the information systems themselves—especially with respect to identifying and tracking patients as they move between different services and levels of care— as well as improving the management and use of health information for resource allocation, intervention design, and accountability.
	Potential ‘quick wins’ for addressing critical bottlenecks to maternal ART can sometimes be identified and acted on. These could include relaxing treatment protocols to enable initiation of ART at the time of testing regardless of CD4 level, enabling task shifting, queue prioritization, aligning ANC and ART visits, and POC CD4 testing.
	Opportunities to increase the directness, intensity, frequency and extension of the health system's engagement with pregnant HIV-infected women should be identified and pursued. This would include not only patient-provider engagement within the health system itself but also strengthening of the facility/community continuum through the development of coordinating and supporting interventions such as peer mentors, community health workers coordinators, support groups, etc.
	Effective and sustainable interventions to support maternal ART, however, should be multi-pronged and multi-leveled and seek to make an impact across the cascade at both facility and higher levels of the health system.
**Research Gaps and Priorities**	Critical gaps in the evidence base regarding maternal ART include adherence outcomes, and factoring affecting adherence; the role of timeliness and timing as discrete variables; the barriers and enablers for those who never make it to health services and those who drop out, and maternal ART outcomes along the full cascade and using a variety of denominators for different comparative evaluation (e.g. ART program performance with respect to the population in care versus ART program performance with respect to the population in need).
	Program evaluations using strong, prospective research designs in pragmatic settings should be prioritized in order to better characterize likely maternal ART outcomes and challenges in settings outside small pilot interventions.
	Measures of adherence should be standardized to enable comparison across programs and studies.

We have drawn three over-arching conclusions from the findings and analysis presented above. The first is the lack of focus on the unique experiences, needs and vulnerabilities of HIV-infected pregnant and postpartum women. Health systems need to pay more attention to finding ways to facilitate their movement effectively through both maternal health and HIV care. Second, supporting the movement of these women along the maternal ART cascade will require both a thorough diagnosis of the relevant barriers and enablers, as well as systemic, simultaneous interventions along several points of the cascade. This remains critical for the development and implementation of effective integrated HIV and maternal health services.

Finally, there are a number of critical evidence gaps and priorities for future research that merit attention. Only by better understanding the complex and inter-related health system barriers and enablers that determine women's movement through both maternal health and HIV care can we design interventions that will improve maternal ART initiation, retention and adherence. This is essential for ending preventable child and maternal deaths and achieving an AIDS-free generation.

## Supporting Information

Table S1 **Characteristics of Included Studies.**
(DOC)

Table S2 **Strength of Evidence and Generalizability.**
(DOC)

Table S3 **Narrative Synthesis.**
(DOC)

Table S4 **Summary of Interventions and Reported Outcomes.**
(DOC)

Table S5 **PRISMA Checklist.**
(DOC)

Table S6 **Search Strategies.**
(DOC)
