# Supplementary material for: A Systematic Review of Health System Barriers and Enablers for Antiretroviral Therapy (ART) for HIV-Infected Pregnant and Postpartum Women
Source: PLoS One. 2014 Oct 10;9(10):e108150. doi: 10.1371/journal.pone.0108150 (PMC4193745; doi:10.1371/journal.pone.0108150)
Supplement: Table S1 — Characteristics of Included Studies. (DOC) [file pone.0108150.s001.doc]

# Characteristics of Included Studies (Full Table)

| **Study ID #** | **Author (1st)** | **Region** | **Geographic Setting** | **Type of Facility** | **Study Design** | **Sample Size** | **Participants** | **Primary Outcome of Interest** | **Explicit Intervention Tested** | **Treatment Protocol** | **Risk of Bias** | **RoB Notes** |
| --- | --- | --- | --- | --- | --- | --- | --- | --- | --- | --- | --- | --- |
| 1 | Addala | Asia | Unclear | Private hospitals | Retrospective record review | 13,163 | Pregnant women | Initiation | No | Option A | High | Very little methods data in abstract |
| 2 | Awiti-Ujiji | Sub-Saharan Africa | Both | Unknown (names taken from PMTCT registers) | Qualitative | 26 | HIV+ women with infants <6 months old | Adherence | No | Lifelong ART | Low | Strong qualitative design |
| 3 | Ayuo | Sub-Saharan Africa |  | All | Retrospective Record Review | 4284 | HIV+ pregnant women initiating CART b/w Jan 2006-Feb 2009 | Adherence and Retention | No | Option A and Option B | Moderate | Retrospective design, self-report |
| 4 | Barr | Sub-Saharan Africa | Both | Various | Retrospective record review | 19,735 | HIV+ pregnant and breastfeeding women starting on ART | Initation and Retention | No | Option B+ | High | Very little methods data in abstract |
| 5 | Bullo | Latin America | Urban | Hospital | Prospective cohort | 291 | Women in maternity ward | Initiation | No | N/A | Moderate | Prospective design but little methods data |
| 6 | CDC | Sub-Saharan Africa | Both | All | Intervention/ Evaluation | Malawi MOH data | HIV+ pregnant and breastfeeding women starting on ART | Initiation | Yes | Option B+ | Low | Prospective, routine data with high level of DQ monitoring |
| 7 | Chinkonde | Sub-Saharan Africa | Both | Clinics | Qualitative | 40 | HIV+ PMTCT program participants (28, including participants and dropouts) and husbands (12) of some of those women | Retention | No | Option A | Moderate | Good qualitative design, limited range of participants, data collection |
| 8 | Clouse | Sub-Saharan Africa | Urban | District Hospital | Retrospective Record Review | 273 | HIV+ pregnant women | Initiation and Retention | No | Option A | Moderate | Retrospective design, missing data of various sorts |
| 9 | Coria | Latin America | Urban | Clinic | Retrospective Record Review | 473 | HIV+ women who gave birth between 1999 and 2005 | Initiation | No | Option A | High | Retrospectice design, missing data, under-powered |
| 10 | Dean | Sub-Saharan Africa | Urban | Clinics | Intervention/ Evaluation | 7 | HIV+ pregnant women diagnosed with HIV during current pregnancy | Initiation | Yes | Option A | High | Thin mixed methods design |
| 11 | Delvaux | Sub-Saharan Africa | Both | Clinics | Qualitative | 236 | HIV+ women (125 who adhered to sdNVP and 111 women who did not) | Adherence | No | sdNVP | Moderate | Case-control design |
| 12 | Duff | Sub-Saharan Africa | Both | Tertiary Hospital | Qualitative | 45 | HIV+ women in PMTCT-Plus client register (including women taking HAART, those who never enrolled, those who enrolled but never began, and those who defaulted) | Initiation | No | Option B | Moderate | Well-done qualitative design, limited in scope, participants |
| 13 | Esiru | Sub-Saharan Africa | Both | Unclear | Retrospective record review | 350 | HIV+ pregnant women | Initiation and Retention | Yes | Option A | Moderate | Abstract, retrospective review, some design notes |
| 14 | Ferguson | Sub-Saharan Africa | Urban | Tertiary Hospitals | Retrospective Record Review | 1,129 | HIV+ women diagnosed during pregnancy between 2008-2010 | Initiation and Retention | No | Option B | Moderate | Retrospective |
| 15 | Jerome | Latin America | Urban | Tertiary Hospital | Qualitative | 18 | HIV+ women who had given birth to at least 1 child with knowledge of their status | Retention | No | Unclear…B+? | Moderate | Well-done qualitative design, some bias issues in methods |
| 16 | Kasenga | Sub-Saharan Africa | Rural | District (Church owned hospital, funded by USAID) | Qualitative | Unclear | HIV+ women in PMTCT program, health care workers, community members, traditional birth attendants | Initiation | No | sdNVP | High | Very little methods data in summary |
| 17 | Killam | Sub-Saharan Africa | Urban | Clinics | Intervention/ Evaluation | 37,203 | Women attending ANC between July 2007 and July 2008 | Initiation and Retention | Yes | Option A | Low | Strong prospective design |
| 18 | Kirsten | Sub-Saharan Africa | Rural | District Hospital | Prospective cohort | 122 | HIV+ pregnant women eligible for PMTCT prophylaxis | Adherence | No | Option A | Low | Prospective design, fairly well controlled conditions |
| 19 | Kuonza | Sub-Saharan Africa | Both | District Hospital | Prospective cohort (Qualitative) | 212 | HIV+ mothers in postnatal PMTCT program using PMTCT prophylaxis | Adherence | No | sdNVP | High | Cross-sectional, self report |
| 20 | Mandala | Sub-Saharan Africa | Both | Clinics | Retrospective Record Review | 14,815 | HIV+ pregnant women registered in PHCs from Apr 2007-Mar 2008 | Initiation | No | sdNVP, Option A and Option B | Low | Prospective design, large sample, well controlled |
| 21 | Mellins | North America | Both | Tertiary Hospitals | Retrospective Record Review | 396 | HIV+ pregnant and postnatal women (6 mnths postpartum) in March 2001-June 2005 | Adherence | No | Lifelong ART | Moderate | Prospective design, self report |
| 22 | Mirkuzie (a) | Sub-Saharan Africa | Urban | PHCs and private hospitals | Prospective cohort | 282 | HIV+ mothers | Initation | No | Option A | Low | Prospective, reliability controlled |
| 23 | Mirkuzie (b) | Sub-Saharan Africa | Urban | Hospitals and clinics | Retrospective Record Review | 663,603 | Women attending ANC in health facilities offering PMTCT from Feb 2004-Aug 2009 | Initiation | No | sdNVP and Option A | Moderate | Retrospective design, fairly complete dataset, some control for missing data |
| 24 | Muchedzi | Sub-Saharan Africa | Urban | Clinics | Prospective cohort (Qualitative) | 147 | HIV+ pregnant and postnatal women referred for lifeling ART from PMTCT | Initiation | No | Option A | High | Cross-sectional, self report, response rate |
| 25 | Myer | Sub-Saharan Africa | Urban | Clinic | Prospective cohort (Data and interviews) | 221 | HIV+ pregnant women | Initiation | Yes | Option A | Moderate | Well designed and controlled but small sample and time frame |
| 26 | Myer | Sub-Saharan Africa | Urban | Clinic | Retrospective Record Review | 490 | HIV+ pregnant women referred to ART clinic | Initiation | No | Option A | Moderate | Retrospective design. Fairly well controlled |
| 27 | Nassali | Sub-Saharan Africa | Urban | Tertiary Hospital | Prospective cohort | 289 | HIV+ mothers in postnatal PMTCT program | Retention | No | PMTCT | Moderate | Mixed methods with some prospective component and validity controls |
| 28 | Ngidi | Sub-Saharan Africa | Both | Hospitals and clinics | Intervention/ Evaluation | District data | HIV+ pregnant women eligible for lifelong ART | Initiation | Yes | Lifelong ART (Option A) | Low | Controlled, non-randomised trial |
| 29 | O'Gorman | Sub-Saharan Africa | Rural | N/A | Qualitative | 70 | Antenatal and postnatal women, fathers, TBAs, health workers, grandmothers, community leaders | Initiation | No | sdNVP | Moderate | Limited qualitative design but wide range of participants |
| 30 | Pai | Asia | Rural | Tertiary Hospital | Intervention/ Evaluation | 1002 | Pregnant women testing for HIV, syphilis and hepatitis B thorugh triple point-of-care testing (STPOC) | Initiation | Yes | N/A | Moderate | Prospective study but sampling and control weaknesses |
| 31 | Peltzer | Sub-Saharan Africa | Rural | Clinics | Prospective cohort | 815 | HIV+ women with a baby 3-6 months old | Adherence | No | sdNVP | High | Cross-sectional, self report |
| 32 | Peltzer | Sub-Saharan Africa | Unclear | Clinics | Prospective cohort | 746 | HIV+ antenatal women at 28 wks gestation or more (139) and HIV+ postnatal women with infant aged 1 week to 12 months (607) | Adherence | No | Option A for PMTCT | High | Cross-sectional, self report |
| 33 | Ramers | Sub-Saharan Africa | Unclear | Clinics | Retrospective record review | 57,210 | Women registered at ANC sites (seven) | Initiation | Yes | Option A | Moderate | Well-designed routine intervention study but limited methods data in abstract |
| 34 | Sprague | Sub-Saharan Africa | Both | Tertiary and regional hospitals, clinic | Qualitative | 153 | Informants include 83 HIV+ women, 32 caregivers of HIV+ children, 38 key informants (HIV and PH specialists, academics, nurses, doctors& counsellors) | Initiation | No | Option A | Moderate | Standard qualitative design but good sample size and triangulation |
| 35 | Stinson | Sub-Saharan Africa | Unclear | Clinics | Retrospective record review | 14,987 | Women registered at ANC sites (four) | Initiation | Yes | Option A | Moderate | Retrospective design, good HIS systems, small cluster sample size |
| 36 | Tsague | Sub-Saharan Africa | Both | District hospitals and health centers | Retrospective record review | 40,674 | Pregnant women who visited ANC | Initiation | Yes | Transition from sdNVP to Option A and Option B | Moderate | Retrospective design, good HIS systems |
| 37 | Tshabalala | Sub-Saharan Africa | Unclear | Unclear | Unclear | Unclear | HIV+ pregnant women | Initiation | Yes | Option A | High | Very little methods data in abstract |
| 38 | Van Schalkywk | Sub-Saharan Africa | Both? | Tertiary Hospital | Retrospective record review | 250 | HIV+ pregnant women who initiated HAART | Initiation | No | Option A | High | Retrspective design, tertiary setting, small sample |
| 39 | Varga | Sub-Saharan Africa | Rural and semiurban | Clinic catchment areas | Qualitative | Unclear, 140+ in FGDs and survey | HIV+ adolescent mothers aged 15-19 who had been through ANC in PMTCT pilot sites, clinic staff near to adolescent age, and young adults working on local SRH adol programs (key informant) | Initiation | No | sdNVP | Low | Strong, well-documented qualitative design |
| 40 | Watson-Jones | Sub-Saharan Africa | Urban | District hositals | Prospective cohort | 403 and 30 | HIV+ pregnant women and ANC and maternity ward helath workers | Initiation | No | Option A | Low | Mixed methods with some prospective component and validity controls |
| 41 | Weigel | Sub-Saharan Africa | Urban | Tertiary and regional hospitals | Retrospective record review | 942 | HIV+ pregnant women | Initiation and Retention | Yes | Option A | Low | Strong prospective design |
| 42 | Winestone | Sub-Saharan Africa | Rural | Unclear | Qualitative | 36 | Healthcare providers | Initiation and Adherence | No | N/A | High | Thin qualitative study and very little methods data in abstract |
