# Supplementary material for: A Systematic Review of Health System Barriers and Enablers for Antiretroviral Therapy (ART) for HIV-Infected Pregnant and Postpartum Women
Source: PLoS One. 2014 Oct 10;9(10):e108150. doi: 10.1371/journal.pone.0108150 (PMC4193745; doi:10.1371/journal.pone.0108150)
Supplement: Table S2 — Strength of Evidence and Generalizability. (DOC) [file pone.0108150.s002.doc]

# Strength of Evidence and Generalizability

Assessments of the strength of the evidence supporting a finding would be guided by responses to the following four questions:

1. What is the strength of the underlying study design in the relevant hierarchy of evidence?
2. What is the risk of bias in the study methodology?
3. What level of detail/context is provided in order to allow interpretation and assessment of individual findings?
4. How frequently is the finding reported in the evidence base?

For each key review finding, we asked two further questions regarding generalizability:

1. How many of the studies supporting this finding came from routine settings (i.e. conducted in existing services)?
2. How many of the studies supporting this finding came from LMICs with high HIV prevalence?

| **Key Review Finding** | **Strength of Evidence Summary** | **Generalizability of Evidence Summary** |
| --- | --- | --- |
| 1a | Regarding integration, evidence is mixed. Four studies with low to moderate risk of bias. Two with strong prospective designs. Three with sufficient detail to interpret. Two with no major differences, two with significant differences between models. Regarding multiplicity of other model factors, wide ranging support from many studies | Regarding integration, two from routine settings without specific support and two from large, well-supported interventions, albeit in routine settings. Regarding multiplicity of other factors, found in many settings, including routine and HIV prevalence. Diversity of model factors included in the finding, however, reduces generalizability. |
| 1b | Only a few studies of varying quality reported this finding directly and most were second-order interpretations from discussion sections or articles. Implicit support for the finding was more wide ranging. | This finding was reported in a wide variety of contexts and seems to be a commonly held perception. |
| 1c | Reported by several studies, most with a very low risk of bias, from different settings and regions. Sufficient detail provided to interpret. | Reported in several geographic regions (unusual) and different settings. |
| 1d | Reported in many studies. Most reported significant dropout. Qualitative studies detailed a range of barriers to service integration and a wide range of quantitative studies both assessed dropout and tested for the relevant health systems barriers (e.g. model of care, availability of POC CD4). | This finding was reported across a wide range of contexts, and was particularly common in studies conducted in pragmatic settings. |
| 2a | Many studies reported this finding in both first and second-order interpretations, many of them well-designed. The qualitative studies provided rich detail with respect to the nature of the communication and coordination problems. | This finding was reported across a wide range of contexts, and was particularly common in studies conducted in routine settings. Well-supported interventions also targeted these barriers. |
| 2b | Comes from several studies of varying quality. CD4 testing as a barrier was a strong sub-theme but the others are less strong. Often not a lot of detail presented to interpret and comes from both first and second order findings. | Many elements of the finding were found in routine settings with high HIV prevalence and weak health systems. |
| 2c | Reported by a few studies as important. More often in second order interpretations (discussion sections). Not much detail provided. Sub-theme around confusion regarding protocols and referral procedures was strongest element. | Finding was reflected in several studies in routine settings with weak health systems. Consistent with other barriers reported from these settings. |
| 3a | Reported by many studies of varying quality. Not always clear whether resource constraints or poor management are behind some of the barriers. Fair amount of detail on how these barriers affect pregnant women more than other patients. | Finding was reflected in many studies in routine settings with weak health systems. Consistent with other barriers reported from these settings. |
| 3b | Reported in only a few studies, mostly in second order interpretations. Few studies address system-level issues. Weak health information systems the most consistent sub-theme. | Finding was reflected in a few studies in routine settings with weak health systems. Consistent with other barriers reported from these settings but little detail on context. |
| 4a | Widely reported in many studies, especially qualitative studies with both high and low risk of bias. Rich detail often offered. Reported in less detail in quantitative studies but with consistent effects. | Finding was reported in many studies across a wide range of settings and geographic regions. |
| 4b | Found in several studies with low risk of bias but often with not much detail. Found in both first and second order interpretations. | Found in several studies but little detail on context and varying effects of the factors listed. |
| 5a | Though several studies described interventions that were often quite different and could this not be compared, the studies were consistent and strong with respect to this finding. Several of the studies were low risk of bias and rich in detail. | Finding was reported in several studies in a range of routine settings with weak health systems. Rich detail was available in several of these studies. |
