# Supplementary material for: A Systematic Review of Health System Barriers and Enablers for Antiretroviral Therapy (ART) for HIV-Infected Pregnant and Postpartum Women
Source: PLoS One. 2014 Oct 10;9(10):e108150. doi: 10.1371/journal.pone.0108150 (PMC4193745; doi:10.1371/journal.pone.0108150)
Supplement: Table S3 — Narrative Synthesis. (DOC) [file pone.0108150.s003.doc]

# Narrative Synthesis (adapted from Popay et al.)

| **MAIN ELEMENTS OF 4 STAGE NARRATIVE SYNTHESIS** | **PURPOSE** | **TOOLS AND FRAMEWORKS USED** |
| --- | --- | --- |
| **1. Developing a theoretical model of how the interventions work, why and for whom** | - To inform decisions about the review question and what types of studies to review - To contribute to the interpretation of the review’s findings - To assess how widely applicable those findings may be | WHO Building Blocks Model  SURE Framework  Review Scoping Discussion among team members |
| **2.Developing a preliminary synthesis** | - To organise findings from included studies in order to:   - Identify and list the facilitators and barriers to implementation reported   - Explore the relationship between reported facilitators and barriers | Thematic analysis |
| **3. Exploring relationships in the data** | - To consider the factors that might explain any differences in the facilitators and/or barriers to successful implementation across included studies - To understand how and why interventions have an effect | Comparative case analysis |
| **4. Assessing the robustness of the synthesis product** | - To provide an assessment of the strength of the evidence for drawing conclusions about the facilitators and/or barriers to implementation identified in the synthesis. Generalising the product of the synthesis to different population groups and/or contexts | Narrative assessment guided by 4 questions about strength and 2 questions about generalizability |
