# Supplementary material for: A Systematic Review of Health System Barriers and Enablers for Antiretroviral Therapy (ART) for HIV-Infected Pregnant and Postpartum Women
Source: PLoS One. 2014 Oct 10;9(10):e108150. doi: 10.1371/journal.pone.0108150 (PMC4193745; doi:10.1371/journal.pone.0108150)
Supplement: Table S4 — Summary of Interventions and Reported Outcomes. (DOC) [file pone.0108150.s004.doc]

# Summary of Interventions and Reported Outcomes

| **Intervention Type** | **Specific Intervention (Study)** | **Intervention Description** | **Reported Mechanisms/ Effects (Quantitative)** | **Reported Mechanisms/ Effects (Qualitative)** |
| --- | --- | --- | --- | --- |
| Change in Treatment Protocol | Option B+ (CDC 2013) | Roll-out of Option B+ in Malawi. Involved not only revision to the treatment protocol but also integration of ART into ANC services, training of almost all HCWs, task shifting that allowed non-physician ART initiation, and consistent use of EFV for all adult ART first-line, including pregnant women. Also includes quarterly support to every PMTCT/ART site in the country by a national/int’l supervision team, new patient registers allowing for cohort analysis, new HIV program reporting | 748% increase in the number of pregnant or breastfeeding women starting ART. Maternal ART initiations went from 5% of all initiations to 35%. 77% retention after 12 months, similar to adult ART retention rate. | Removing need for CD4 counts, removing travel between ANC and ART services, allowing task shifting, reducing number of steps in cascade. |
| mHealth and Peer Support | SMS Support Group  (Dean et al. 2012) | Interactive SMS support group with 6-10 participants, facilitated by 1) an HCW with access to HIV and OBGYN technical advice, and 2) a peer mentor with PMTCT experience | Half of the questions asked were regarding medical information. Other half was psychosocial dialogue. | Well-received by participants but they had high expectations of quick physician response. Technical problems, though limited, had marked impact on level of conversation. |
| Fast-Tracking | Rapid Initiation of ART in Pregnancy  (Myer et al. 2013) | Rapid Initiation of Antiretroviral Therapy in Pregnancy (RAP) program at one ANC clinic. Involved same-day POC CD4 testing (ART enrollment <=350), counseling by lay health worker, psychological and substance abuse screening, clinical evaluation, and ART initiation in the absence of major medical or psychological co-morbidities. Follow-up after 1 week and thereafter in sync with ANC visit schedule. | 91% of those starting ART started on the same day. Initiation rate reported to be much higher than estimated rate in same setting. Short delays due to medical concerns, longer delays due to psychosocial concerns. | High level of acceptability among women, and motivation to accept for baby’s health. No direct comparison/control and no direct assessment of health systems factors but intervention implies key factors at work (POC CD4, same-day counseling and clinical workup). |
| Fast-Tracking | Campaign among HCWs to speed up ART initiation  (Ngidi et al. 2013) | Six-month campaign among HCWs (“Masibavikele”) using quality improvement principles to fast-track ART for pregnant women. Four principles: galvanizing and time-bound aim, evidence-based interventions, social system for organizing participation, and clear outcomes measurement framework. Included management and information systems strengthening, simplifying ART initiation, creating roving ART initiation teams. | Monthly ART referral rate among eligible women increased from 58.6% to 76.9%. No significant change in control district.  Monthly ART initiation rate among referred women increased from 55.4% to 65.8%. No significant change in control district. | Changes were sustained six months post intervention. Intervention implies the relevant health system factors, including management leadership, publicity/awareness, training of HCWs, improved data use and accountability, streamlining ART cascade, mobile ART initiation. |
| Point-of-care Testing | Simultaneous triple point-of-care testing  (Pai et al. 2012) | Simultaneous triple point-of-care testing for HIV, syphilis and hepatitis B. Combined pre- and post-test counseling and POC testing for the 3 diseases and referral for treatment/prophylaxis. | 98% consented to testing, 96% of these completed testing. Approx. 45 minutes per patient. Only 9% of patients had been screened for all three previously. High acceptability. | Test of feasibility and acceptability of STPOC testing. No major concerns noted. |
| HIV/ANC Integration | Integrated, proximal, and distal models of care  (Stinson et al. 2010) | Comparison of three service delivery models for linking ART and ANC services. The ‘integrated’ model offered ART/ANC care once a week by a doctor. The ‘proximal’ model referred women to an ART service in a separate building but on the same site. The ‘distal’ model referred women to ART services within 5km. All sites used the same protocols for ANC care and HIV testing | No significant differences in ART initiation before delivery by model (55%, 48%, 47%). No significant differences in time on ART before delivery by model. No longer-term outcomes measured. | Substantial barriers remain to coordinating ANC and ART, even when integrated. Few doctors available and nurses not allowed to initiate ART. Drug readiness training was 4-6 weeks. |
| HIV/ANC Integration | Full package versus stand-alone models of care  (Tsague et al. 2010) | Comparison of two service delivery models for linking ART and ANC services. The ‘stand-alone’ sites provided PMTCT care alone (including dual ARV and sdNVP) and the ‘full package’ sites offered PMTCT as well as ART care (including both short-course and lifelong ART). | CD4 testing 30% more likely at full package sites. Enrolment in ART services twice as likely at full package sites. However, no difference with respect to initiation among eligible women between the two types of sites. | Initiation between site types was both similar and at a high rate (85%), indicating that broader health systems improvements in CD4 testing, training and patient follow-up may have erased the effect of differences between the site types earlier in the cascade. |
|  | Decentralization of HIV Care into local ANC clinics (Ramers 2010) | Integration of HAART into routine ANC services at local facility level. | Before integration, 3.3% of HIV+ women initiated ART during pregnancy, after integration, 7.6% did so (P = < 0.0001). |  |
|  | Integration of HIV into ANC Services (Killam 2010) | Integration of HAART into routine ANC services at local facility level. | Twice as many women enrolled in and initiated treatment in the integrated services. |  |
| Multi-Layered Support to PMTCT/ART Programs | Quailty improvement intervention to improve PMTCT (Tshabalala 2010) | Quality improvement strategies included training of health care workers on quality improvement (QI) methods, setting goals, forming QI teams, process mapping to identify gaps in the system, initiating plan-do-study-act cycles, data management, employing a quality mentor to provide technical assistance, monthly data review meetings and quarterly QI learning sessions |  | Increased the demand for PMTCT services; more pregnant women tested for HIV and more accessed ART |
| Multi-Layered Support to PMTCT/ART Programs | Comprehensive maternal ART support intervention (Esiru 2013) | A programmatic package targeting loss at each point in the cascade. included new patient tracking registers and referral mechanisms, rationalized clinic systems, new logistics systems, and a new training-mentorship model. | 500% increase in completion of the PMTCT cascade (including maternal ART outcomes). Increase in post-delivery retention from 12% to 39%. |  |
|  | Comprehensive maternal ART support intervention  (Weigel et al. 2012) | Wide range of interventions put in place over three years to better link ART and ANC services. Initiation of routine CD4 testing among pregnant women identified as HIV infected at 1st ANC visit, PITC, paper-based system using triplicate referral slips to monitor completeness of referral, vehicle available to transport eligible pregnant women from ANC to ART, new clinic to provide comprehensive HIV care within 200 m, identification of ART link person to ANC, revised referral letter from ANC⁄ PMTCT to ART, ‘PMTCT women’ prioritised at reception, no guardian required to start ART same day, signposts to mark the way from ANC to ART, introduction and use of an electronic data system (EDS) for patient care, all women attending ANC get a bar-coded label with a unique hospital ID. | Reduction in time from ART referral to ART initiation from 19 days to 0 days between 2006 and 2009. Retention at six months increased from 17% in 2006 to 65% in 2009. Percentage starting ART while pregnant increased from 17% in 2006 to 74% in 2009. | Physical distance, even of 200m, can present a stubborn obstacle to referral. Delay between eligibility and ART initiation is critical and preventable. Prioritizing women in queues and removing other obstacles to initiation (like requiring a guardian be present) were also important aspects. Finally, importance of robust M&E systems for tracking patients and evaluating services. |
