# Supplementary material for: A Systematic Review of Health System Barriers and Enablers for Antiretroviral Therapy (ART) for HIV-Infected Pregnant and Postpartum Women
Source: PLoS One. 2014 Oct 10;9(10):e108150. doi: 10.1371/journal.pone.0108150 (PMC4193745; doi:10.1371/journal.pone.0108150)
Supplement: Table S6 — Search Strategies. (DOC) [file pone.0108150.s006.doc]

# Search Strategies

## Full search strategy for PubMed

((((pregnant OR partum OR natal OR breastfeed* OR mother OR maternal)) AND HIV) AND (ARV OR ART OR haart OR pmtct OR antiretrovir* OR treatment)) AND (retention OR adherence OR compliance OR initiation OR ltfu OR loss to follow up))

Dates: 2008 (inclusive) to present

## List of Gray Literature Sources Reviewed

| **Source** | **Number of Documents Screened** |
| --- | --- |
| United Nations Joint Program on HIV/AIDS (UNAIDS) | 142 |
| World Health Organization (WHO) | 224 |
| United States Agency for International Development (USAID) – Development Experience Clearinghouse (DEC) | 303 |
| ICAP, Columbia University | 26 |
| Elizabeth Glaser Pediatric AIDS Foundation (EGPAF) | 66 |
| Pathfinder International | 10 |
| International AIDS Society (IAS) | 620+ |
| Conference on Retroviruses and Opportunistic Infections (CROI) | 100+ |
| International Society For Sexually Transmitted Diseases Research (ISSTDR) | 95 |
| International Conferences on Improving Use of Medicines (ICIUM) | 50+ |
